# Too much of a good thing? Hand hygiene and the long-term course of contamination-related obsessive-compulsive symptoms

**DOI:** 10.3389/fpsyg.2024.1279639

**Published:** 2024-03-08

**Authors:** Lena Jelinek, Anja S. Göritz, Franziska Miegel, Lea Schuurmans, Steffen Moritz, Amir H. Yassari, Jana Christina Müller

**Affiliations:** ^1^Department of Psychiatry and Psychotherapy, University Medical Center Hamburg-Eppendorf, Hamburg, Germany; ^2^Behavioral Health Technology, University Augsburg, Augsburg, Germany

**Keywords:** mental health, public health, SARS-CoV-2, C19, hygiene, safety behavior

## Abstract

Increased hygiene behavior may be a factor in the development of contamination-related obsessive-compulsive symptoms (C-OCS). We aimed at investigating (1) the course of C-OCS over 1 year after the start of the COVID-19 pandemic and (2) the effects of changes in hand hygiene (i.e., duration and frequency of handwashing) and related distress regulation on the long-term course of C-OCS. In a longitudinal study, we assessed 1,220 individuals from the German general population at the start of the COVID-19 pandemic (t1), 3 months later (t2), and 12 months later (t3). Pre-pandemic data were available in a subsample from 2014 (*n* = 430). A decrease in C-OCS over the first year of the pandemic emerged with a small effect size. Thirty-six percent of the participants scored above the clinical cut-off score at t1, 31% at t2, and 27% at t3. In 2014, only 11% scored above the clinical cut-off score. Hierarchical regression showed that C-OCS at t1 was the strongest predictor of a long-term increase in C-OCS. With small effect sizes, change in the duration (not frequency) of handwashing from t1 to t2, as well as the distress-reducing effect of handwashing served as additional predictors. Implications for information on hand hygiene guidelines are discussed.

## 1 Introduction

During the COVID-19 pandemic, the world’s population was subjected to restrictive measures by governments (e.g., Mækelæ et al., 2020; Aknin et al., 2022; Gennaro et al., 2023) to limit the spread of the virus. Among these anti-contagion measures, handwashing was omnipresent and handwashing protocols were strongly advocated (e.g., by the World Health Organization [WHO]). As in previous pandemics, research focused on how to promote adherence to hygiene protocols.

In patients with contamination-related obsessive-compulsive disorder (C-OCD), hygiene rituals (e.g., long or frequent ritualized handwashing) and avoidance behavior (e.g., social distancing, wearing gloves or face masks) serve to reduce unpleasant feelings (e.g., anxiety, disgust, guilt) evoked by obsessions (intrusive thoughts, e.g., “I could get infected and die,” “I could infect someone else”) and may thus be considered a strategy to regulate distress. According to early behavioral models (Mowrer, 1939; Dollard and Miller, 1950) that influenced first-line treatment of OCD (i.e., exposure and response prevention), the reduction of distress reinforces the hygiene behavior and thereby maintains and increases OCS in the long term (Salkovskis, 1999). The ability to stop handwashing, not the initial perception of threat of contamination, differentiates individuals without obsessive-compulsive disorder and individuals with checking compulsions from individuals with C-OCD (Hinds et al., 2012). Moreover, criteria for terminating washing rituals are subjective (“until it feels right,” p. 143, Wahl et al., 2008) in people with C-OCS compared to people with obsessive-compulsive disorder other than C-OCD. Handwashing in people with high contamination fear is characterized by the prolongation—not by the repetition—of handwashing compared to the control participants (Dean and Purdon, 2021).

Adherence to hygiene protocols (i.e., increased handwashing) at the start of the COVID-19 pandemic may thus have provided distress relief, in addition to the intended infection prevention, thus promoting an increase in contamination-related obsessive-compulsive symptoms (C-OCS, e.g., Shafran et al., 2020; Ornell et al., 2021). Hygiene behavior has been cross-sectionally related to C-OCS (Samuels et al., 2021) and anxiety (Newby et al., 2020) in the general population. However, although evidence of an increase in obsessive compulsive symptoms in general at the start and over the early course of the pandemic is accumulating for the general population and clinical samples with OCD (Grant et al., 2021; Guzick et al., 2021; Zaccari et al., 2021; Linde et al., 2022), findings on the course of C-OCS are mixed (Alonso et al., 2021; Fontenelle et al., 2021; Khosravani et al., 2021; Jelinek et al., 2021a,b; Hezel et al., 2022; Moreira-de-Oliveira et al., 2022; Otte et al., 2023). Investigating an interval of 8 months, Grøtte et al. (2022) reported that while C-OCS was still elevated in a large population-based sample (*N* = 3,405), the C-OCS trend declined from April to December 2020. As in previous studies (Fontenelle et al., 2021; Jelinek et al., 2021a; Alonso et al., 2021), the predictor with the largest effect sizes for the long-term course of C-OCS was the baseline level of C-OCS (β = 0.44). Adherence to COVID-19 guidelines was also investigated in April 2020 as a predictor, including not only adherence to recommendations regarding hand hygiene but also other pandemic measures (such as doing office work at home and avoiding travel). Adherence to COVID-19 guidelines in April 2020 served as a significant predictor of C-OCS in December 2020, but effect sizes were small (β = 0.06) and the effects of handwashing in particular as well as its potential distress-regulating effect were not investigated.

A connection between hand hygiene and such distress-reducing effect (reinforcement) can be assumed as experimental work in a student sample (Deacon and Maack, 2008) has shown that an increase in contamination-related safety behavior (including handwashing) increases contamination-related fears. Furthermore, models of OCD (see above and Dollard and Miller, 1950; Salkovskis, 1999) emphasize the role of reinforcement in the development of the disorder.

Our aim in the current study—to improve our understanding of the development of C-OCD—was twofold. First, we aimed to investigate the longitudinal course of C-OCS (OCI-R washing subscale) 12 months after the start of the pandemic. Second, we sought to examine the effect of changes in hand hygiene (i.e., duration and frequency of handwashing) and distress regulation (i.e., reinforcement after handwashing) over the first months of the COVID-19 pandemic on the longitudinal course of C-OCS beyond established predictors such as age, educational level, and baseline scores of contamination-related OCS (Abba-Aji et al., 2020; Fontenelle et al., 2021; Jelinek et al., 2021a; Alonso et al., 2021; Grøtte et al., 2022). Building on previous cross-sectional results in individuals with contamination fears (Dean and Purdon, 2021), on behavioral models of OCD (Dollard and Miller, 1950; Salkovskis, 1999), as well as experimental studies with student samples (Deacon and Maack, 2008), we expected that an increase in the duration rather than the frequency of handwashing and increased negative reinforcement after handwashing (distress reduction) would be associated with a long-term increase in C-OCS in the general population.

## 2 Materials and methods

### 2.1 Recruitment and procedure

As previously described (Jelinek et al., 2021a), we invited *N* = 14,285 members of WisoPanel® at www.wisopanel.net to participate in a web-based assessment (the OCI-R) between March 21 and March 30, 2020 (Jelinek et al., 2021a). WisoPanel® includes people from the general population in Germany (Piccolo and Colquitt, 2006; Göritz, 2007). Göritz (2014) provides a description and methodological inquiry into WisoPanel®. Göritz et al. (2021) obtained superior data quality in WisoPanel® compared to two crowdsourced samples

At the time of the assessment, the first COVID-19-related lockdown had been announced in Germany. Of the invitees, *n* = 2,727 [19%] accessed the survey. The answers of *n* = 2,255 participants were included in previous analyses of the data set [participants who did not complete the Obsessive-Compulsive Inventory-Revised (OCI-R) washing subscale, exhibited stereotypical answer patterns, or reported responding untruthfully were excluded (i.e., people indicating in the final question that they had answered the questions untruthfully, see Jelinek et al., 2021a)]. These participants were invited to participate in a second assessment 3 months later between June 22 and June 30, 2020 (t2), soon after the easing of the first lockdown restrictions in Germany. Findings from the data collected at the start of the pandemic (t1) and (t2) have been reported before, focusing on the course of OCS in the general population from t1 to t2 (Jelinek et al., 2021a) and the role of a cognitive bias (unrealistic pessimism) at the start of the pandemic (Jelinek et al., 2022). The focus of the current study was the long-term course of C-OCS. For this reason, participants were assessed again 12 months later, between April 3 and April 12, 2021 (t3). During t3, Germany was not under lockdown, but restrictions were still in place, including the “federal emergency brake” if the 7-day incidence was more than 100 in any given region (leading, e.g., to a curfew and store opening restrictions). As previously reported (Jelinek et al., 2021a), a subsample was assessed with the OCI-R between March 30 and April 7, 2014 (pre-pandemic data) as part of a study on adaptive and maladaptive coping styles (Moritz et al., 2016).

For all assessments, we used the online platform Unipark/Questback® (Globalpark AG).

### 2.2 Assessment

#### 2.2.1 C-OCS

At t1, t2, and t3, we assessed C-OCS using the washing subscale of the German version of the Obsessive-Compulsive Inventory-Revised (OCI-R) (Foa et al., 2002; Gönner et al., 2008). The OCI-R has been shown to be sensitive to change (Abramowitz et al., 2005), and psychometric properties are good for the German version of the questionnaire (Gönner et al., 2007, 2008). In the present study, internal consistency of the OCI-R washing subscale was acceptable with Cronbach’s *α* = 0.70 at t1, *α* = 0.73 at t2, and *α* = 0.75 at t3. A clinical cut-off score of 3 for the washing subscale has been reported for the German version of the OCI-R as sensitivity (0.98) and specificity (0.91) were best at this cut-off score (Gönner et al., 2009).

#### 2.2.2 Hygiene behavior

At t1 and t2, we asked about the duration of hygiene behavior [“In the last 7 days, how long did you wash your hands on average?” on a seven-point scale: (1) “Did not wash my hands at all,” (2) “< 10 s,” (3) “10 s to <20 s,” (4) “20 s to <30 s,” (5) “30 s to <60 s,” (6) “1 min to <2 min,” (7) “2 min or longer”] and frequency of handwashing [“In the last 7 days, how often did you wash your hands each day?” on a seven-point scale: (1) “Not at all,” (2) “1 time,” (2) “2 times,” (3) “3 times,” (4) “4 times,” (5) “5 times,” (6) “6 times,” (7) “More than 6 times”]. Furthermore, we assessed the extent of reinforcement after handwashing by asking which answer was most applicable on a four-point scale following “After I wash my hands”: (1) “I feel unchanged,” (2) “I have a slightly better feeling than before,” (3) “I feel relieved,” or (4) “I have a much better feeling than before.”

#### 2.2.3 Data analysis

The main analyses were conducted with IBM SPSS® Statistics version 26, including all available data (complete cases). To investigate change over time, we used paired sample *t*-tests (OCI-R Washing subscale) or the Wilcoxon test (hygiene behavior). Additionally, we calculated repeated measures ANOVAs across the three assessments (t1, t2, t3). To obtain an estimate of whether the prevalence of clinically relevant washing behavior (above the cut-off score for the washing subscale) increased from before the pandemic to 12 months into the pandemic, we planned to demographically compare the percentage of people scoring above the clinical cut-off score of 3 to data assessed in a subsample in 2014.

To investigate predictors of change, we calculated multiple hierarchical regression models. Models A1–A5 analyzed change in C-OCS over time (OCI-R washing subscale at t1 minus OCI-R washing subscale at t3) entered as the dependent variable. Demographic background variables (age, gender, education) were entered as the first block of predictors (model A1) and to control for regression to the mean (following previous publications; e.g., Jelinek et al., 2021b, 2022) psychopathology at t1 (OCI-R washing subscale) as the second block of predictors (model A2). Changes in duration of handwashing over the first 3 months of the pandemic (i.e., t1–t2) were entered as the third block (model A3) and frequency of handwashing in the fourth block (A4). Finally, change in negative reinforcement (i.e., t1– t2) after handwashing was entered as a predictor in the fifth block (model A5). Analyses were recalculated (models B1 to B5) with a changed order (negative reinforcement: block B3; duration of handwashing: block B4; and frequency of handwashing: block B5). To check whether the more complex model represents the data better than the simpler model, we successively compared the models to each other in each additional step using a likelihood ratio test (LRT).

As the primary analyses, we planned complete case (CC) analyses. To evaluate the robustness of the results, missing data were estimated by multiple imputation (sensitivity analyses). The analyses were performed with *R* version 4.1.0 (R Core Team, 2021). Missing data were imputed using multivariate imputation by chained equations (MICE) with 50 iterations per imputation set in a total of 50 sets, using the *mice* package (version 3.14, Van Buuren and Groothuis-Oudshoorn, 2011) and based on the assumption that data were missing at random, conditional on sociodemographic information (e.g., gender, age), and all relevant outcomes across the three assessments. The models were fitted individually in each imputed data set. The model estimates were then pooled by applying Rubin’s Rule (Barnard and Rubin, 1999), using the *mitml* (version 0.4–3, Grund et al., 2021) and *miceadds* packages (version 3.11–6, Robitzsch and Grund, 2021).

As effect sizes, Cohen’s *d* (with *d* ≈ 0.2, *d* ≈ 0.5, and *d* ≈ 0.8, corresponding to small, medium, and large effects) and η_p_^2^ (with η_p_^2^ ≈ 0.01, η_p_^2^ ≈ 0.06, and η_p_^2^ ≈ 0.14, corresponding to small, medium, and large effects) were calculated. Standardized regression weights (β) of 0.1, 0.3, and 0.5 were considered weak, medium, and strong effects, respectively.

## 3 Results

### 3.1 Sample

**Figure 1:**
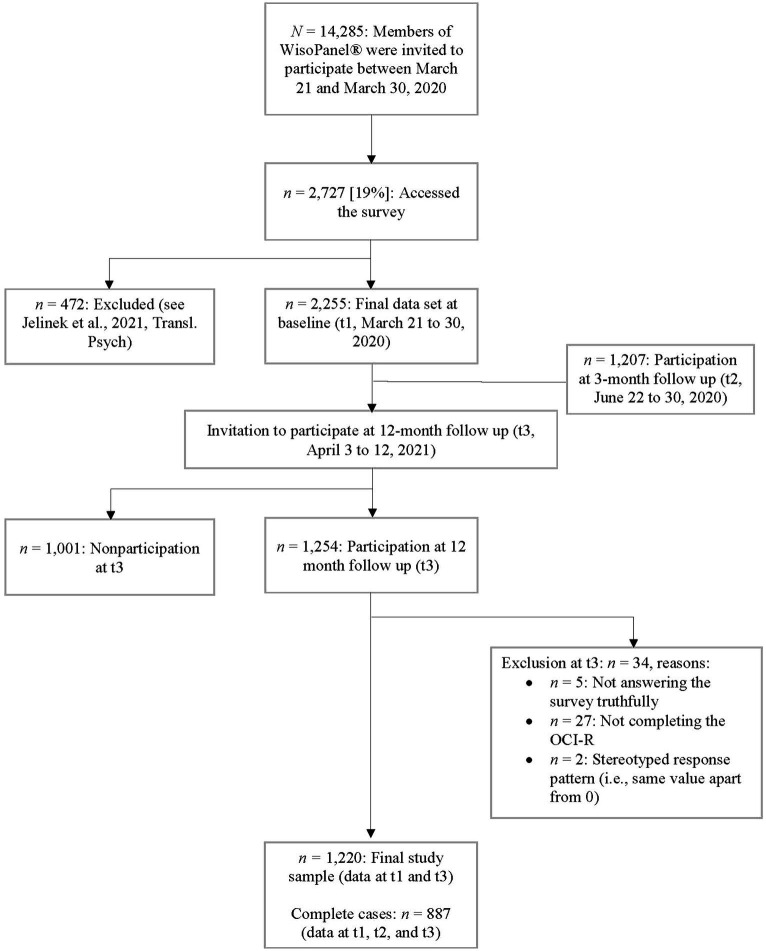
Flow chart.

As can be seen in Figure 1, *n* = 1,254 (55.6%) individuals from the t1 sample participated at t3. Of these, 34 were excluded (see Figure 1 for the reasons), leading to a final sample of *n* = 1,220 (54.1%) with complete data at t1 and t3. On average, responders (those with available data at t1 and t3) were older (*M* = 55.49, *SD* = 13.71) than non-responders (data only available for t1, *M* = 50.85, *SD* = 14.48, *t*[2253] = 7.803, *p* < 0.001, *d* = 0.33) and responders were more likely to be male than female (45.3% vs. 38.2%; χ^2^[1] = 11.793, *p* < 0.001). Responders also had a smaller percentage of participants with A-levels (non-reponders: 65.1% a-levels, χ^2^[1] = 12.128, *p* < 0.001, *d* = 0.20), but they (*M* = 11.92, *SD* = 11.13) had a similar level of OCS (OCI-R total score) to non-responders (*M* = 12.26, *SD* = 10.13, *t*[2228.902] = 0.767, *p* = 0.222, *d* = 0.032). For *n* = 887, data were available for all three assessments (t1, t2, and t3). Of these, OCI-R data were available for *n* = 430 (48.48%) participants from the year 2014.

Demographics for the final sample were as follows: mean age of 54.49 years (*SD* = 13.71), *n* = 667 women (54.7%), and *n* = 707 (85.0%) with an A-level degree (i.e., university entrance qualification).

### 3.2 Longitudinal course of C-OCS

As can be seen in Table 1, C-OCS decreased over time. However, effect sizes were small (Cohen’s *ds* between 0.222 and 0.118, ƞ_p_^2^ = 0.029). When repeated measures ANOVAs were calculated including all three assessments, a significant effect of time with small effect size was found in the complete case (CC) analyses. Multiple imputation (used to correct for missing data) confirmed the results (see Table 1).

**Table 1:** Contamination-related obsessive-compulsive symptoms across time.

	t1 (*n* = 1,220)		t2 (*n* = 891)		t3 (*n* = 1,220)		Paired *t*-tests		Repeated measures ANOVA (t1, t2, t3)
	*M*	*SD*	*M*	*SD*	*M*	*SD*	*t1* vs. *t3*	*t2* vs. *t3*	*MI*
Washing (OCI-R)	2.33	2.32	1.98	2.41	1.81	2.32	*t*(1219) = 7.747, *p* < 0.001, Cohen’s *d* [CI_95%_] = 0.222 [0.165, 0.279]	*t*(890) = 3.529, *p* < 0.001, Cohen’s *d* [CI_95%_] = 0.118 [0.052, 0.184] MI: *t*(1219) = 3.216, *p* = 0.001, Cohen’s *d* [CI_95%_] = 0.100 [0.044, 0.157]	*F*(2, 890) = 26.492, *p* < 0.001, ƞ_p_^2^ = 0.029 MI: *F*(2,1,219) = 34.481, *p* = < 0.001, ƞ_p_^2^ = 0.028

Means and standard deviations were calculated for study completers. OCI-R, obsessive-compulsive inventory-revised; MI, multiple.

At t1, *n* = 322 (36.3%) scored above the clinical cut-off score for the washing subscale, at t2 *n* = 273 (30.8%), and at t3 *n* = 243 (27.4%). Of the 430 participants already assessed in 2014, *n* = 14 (11.2%) scored above the cut-off.

### 3.3 Hygiene behavior

On average, duration (*Z* = −16.77, *p* < 0.001) and frequency (*Z* = −13.67, *p* < 0.001) of handwashing and negative reinforcement (*Z* = −2.18, *p* < 0.001) after handwashing decreased over the first months of the COVID-19 pandemic.

### 3.4 Prediction of the longitudinal course of C-OCS (12 months)

When multiple hierarchical regression models were calculated to predict change in C-OCS during the pandemic, age was a statistically significant predictor in the first step. Older age was associated with a higher increase in C-OCS (see Table 2). When, in the second step, C-OCS at t1 was entered into the model, it presented as an additional significant predictor, indicating that a higher OCI-R washing subscale score at the start of the pandemic was associated with a larger decrease in C-OCS over time. When, in the third and fourth step, changes in handwashing duration and frequency from t1 to t2 were entered, only change in duration was a significant predictor (β = 0.069, *p* = 0.015, ∆*R*^2^ = 0.005), suggesting that a greater decline in handwashing duration during the first 3 months predicted more reduction in C-OCS over the year of follow-up. When change in negative reinforcement after handwashing from t1 to t2 was entered in step 5, increase in negative reinforcement after handwashing served as an additional significant predictor for increase in OCS over time (β = 0.080, *p* = 0.005, ∆*R*^2^ = 0.006). The final model was statistically significant (*R*^2^ = 0.336, *F* = 61.137, *df* = 7, 844, *p* < 0.001), explaining 33.6% of the variance. Switching the order of step 3 and 5 did not essentially alter the results (Supplementary Table S1). Multiple imputation used to correct for missing data largely confirmed the results (Table 3 and Supplementary Table S2). The LRT showed a successive significant improvement in the fit of the model with the increasing addition of the predictors from steps 1 to 3 and from steps 4 to 5. The change from steps 3 to 4 was not significant (Table 2).

**Table 2:** Predictors of change in contamination-related obsessive-compulsive symptoms (outcome: change in OCI − R washing subscale from t1 to t3), *n* = 852.

	*B* [CI_95%_]	β	*p*
**Step 1**	**Model A1**		
Constant	1.531 [−0.721, 2.341]		<0.001
Age	− 0.014 [−0.027, −0.002]	−0.081	0.022
Gender^a^	−0.112[−0.434, 0.209]	−0.024	0.493
A-levels	−0.027[−0.348, 0.295]	−0.006	0.870
**Step 2**	**Model A2**		
Constant	−0.003 [−0.688, 0.682]		0.993
Age	−0.009 [−0.019, 0.002]	−0.048	0.096
Gender^a^	−0.070 [−0.335, 0.196]	−0.015	0.606
A-levels^b^	−0.069[−0.334, 0.196]	−0.015	0.611
OCI − R washing subscale (t1)	0.515 [0.464, 0.565]	0.565	<0.001
χ^2^(1) = 402.51, *p* < 0.001			
**Step 3**	**Model A3**		
Constant	−0.168 [−0.864, 0.528]		0.636
Age	−0.008 [−0.019, 0.002]	−0.047	0.104
Gender^a^	−0.066 [−0.331, 0.198]	−0.014	0.622
A-levels^b^	−0.044 [−0.310, 0.221]	−0.009	0.742
OCI-R washing subscale (t1)	0.516 [0.465, 0.566]	0.566	<0.001
Change in duration of handwashing (t1 − t2)	0.157 [0.031, 0.283]	0.069	0.015
χ^2^(1) = 5.36, *p* = 0.021			
**Step 4**	**Model A4**		
Constant	−0.177 [−0.877, 0.522]		0.619
Age	−0.008 [−0.019, 0.002]	−0.047	0.105
Gender^a^	−0.065 [−0.330, 0.200]	−0.014	0.630
A-levels^b^	−0.045 [−0.310, 0.220]	−0.010	0.739
OCI-R washing subscale (t1)	0.516 [0.466, 0.567]	0.566	< 0.001
Change in duration of handwashing (t1 − t2)	0.155 [0.028, 0.282]	0.068	0.017
Change in frequency of handwashing (t1 − t2)	0.012 [−0.080, 0.103]	0.007	0.801
χ^2^(1) = 0.04, *p* = 0.842			
**Step 5**	**Model A5**		
Constant	−0.187 [−0.884, 0.510]		0.598
Age	−0.008 [−0.018, 0.002]	−0.046	0.109
Gender^a^	−0.069 [−0.333, 0.195]	−0.015	0.609
A-levels^b^	−0.023 [−0.288, 0.241]	−0.005	0.863
OCI-R washing subscale (t1)	0.514 [0.463, 0.564]	0.564	< 0.001
Change in duration of handwashing (t1 − t2)	0.147 [−0.021, 0.274]	0.065	0.023
Change in frequency of handwashing (t1 − t2)	0.003 [−0.088, 0.094]	0.002	0.950
Change in negative reinforcement after handwashing (t1 − t2)	0.198 [−0.060, 0.335]	0.080	0.005
χ^2^(1) = 8.03, *p* = 0.005			

*R*^2^ = 0.008, *F* = 2.216 (*p* = 0.085) for step 1; ∆*R*^2^ = 0.318, *F* = 102.140 (*p* < 0.001) for step 2; ∆*R*^2^ = 0.005, *F* = 83.390 (*p* < 0.001) for step 3; ∆*R*^2^ < 0.001, *F* = 69.425 (*p* < 0.001) for step 4; ∆*R*^2^ = 0.006, *F* = 61.137 (*p* < 0.001) for step 5. b, unstandardized regression coefficient; β, standardized 1 regression coefficient; χ^2^, Chi-Square test statistic for model comparison to the previous step via LRT; OCI-R, Obsessive-Compulsive Inventory-Revised; ^a^1 = male, 2 = female, ^b^0 = no A-level, 1 = A-level and above.

**Table 3:** Results using multiple imputation: predictors of change in contamination-related obsessive-compulsive washing symptoms (outcome: change in OCI-R washing scale from t1 to t3), *n* = 1,220.

	*B* [CI_95%_]	β	*p*
**Step 1**	**Model A1**		
Constant	1.034 [0.434, 1.633]		0.001
Age	- 0.008 [−0.018, 0.001]	−0.049	0.096
Gender^a^	−0.218 [−0.487, 0.051]	−0.046	0.112
A-levels	0.088 [−0.182, 0.358]	0.019	0.525
**Step 2**	**Model A2**		
Constant	−0.581 [−1.101, −0.060]		0.029
Age	−0.001[−0.009, 0.007]	−0.005	0.839
Gender^a^	−0.185 [−0.410, 0.041]	−0.039	0.108
A-levels^b^	0.102 [−0.124, 0.328]	0.022	0.378
OCI-R washing subscale (t1)	0.502 [0.459, 0.546]	0.547	< 0.001
χ^2^(1) = 433.57, *p* < 0.001			
**Step 3**	**Model A3**		
Constant	−0.704 [−1.276, −0.211]		0.009
Age	−0.001 [−0.009, 0.007]	−0.006	0.829
Gender^a^	−0.173 [−0.398, 0.052]	−0.037	0.132
A-levels^b^	0.112 [−0.114, 0.338]	0.024	0.332
OCI − R washing subscale (t1)	0.506 [0.462, 0.549]	0.551	< 0.001
Change in duration of handwashing (t1-t2)	0.142[0.030, 0.254]	0.067	0.013
χ^2^(1) = 6.13, *p* = 0.013			
**Step 4**	**Model A4**		
Constant	−0.743 [−1.276, −0.211]		0.006
Age	−0.001 [−0.009, 0.007]	−0.005	0.829
Gender^a^	−0.171 [−0.396, 0.054]	−0.037	0.136
A-levels^b^	0.108 [−0.118, 0.334]	0.023	0.348
OCI-R washing subscale (t1)	0.507 [0.463, 0.550]	0.552	< 0.001
Change in duration of handwashing (t1-t2)	0.132 [0.019, 0.246]	0.063	0.022
Change in frequency of handwashing (t1-t2)	0.054 [−0.035, 0.142]	0.033	0.234
χ^2^(1) = 1.43, *p* = 0.232			
**Step 5**	**Model A5**		
Constant	−0.764 [−1.295, −0.233]		0.005
Age	−0.001 [−0.009, 0.008]	−0.004	0.878
Gender^a^	−0.182 [−0.406, 0.043]	−0.039	0.113
A-levels^b^	0.128 [−0.097, 0.354]	0.027	0.265
OCI-R washing subscale (t1)	0.504 [0.461, 0.548]	0.549	< 0.001
Change in duration of handwashing (t1–t2)	0.127 [0.014, 0.239]	0.060	0.028
Change in frequency of handwashing (t1–t2)	0.044 [−0.044, 0.132]	0.028	0.322
Change in negative reinforcement after handwashing (t1)	0.180 [0.053, 0.306]	0.074	0.005
χ^2^(1) = 7.82, *p* = 0.005			

*R*^2^ = 0.006, *F* = 2.481 (*p* = 0.059) for step 1; ∆*R*^2^ = 0.297, *F* = 132.275 (*p* < 0.001) for step 2; ∆*R*^2^ = 0.005, *F* = 107.051 (*p* < 0.001) for step 3; ∆*R*^2^ = 0.001, *F* = 89.483 (*p* < 0.001) for step 4; ∆*R*^2^ = 0.006, *F* = 77.769 (*p* < 0.001) for step 5. b, unstandardized regression coefficient; β, standardized regression coefficient; χ^2^, Chi-Square test statistic for model comparison to the previous step via LRT; OCI-R, Obsessive-Compulsive Inventory-Revised; ^a^1 = male, 2 = female, ^b^0 = no A-level, 1 = A-level and above.

## 4 Discussion

The aim of the study was to investigate the longitudinal course of C-OCS 12 months after the start of the COVID-19 pandemic. The study investigated the effect of changes in hand hygiene and related distress regulation on the course of C-OCS in the general population.

Similar to a study from Norway (Grøtte et al., 2022), C-OCS decreased, with a small effect size. This tendency toward a decrease in C-OCS is mirrored by the percentage of scores in the current study above the clinical cut-off score, which fell from 36% (t1) at the start of pandemic to 27% a year later (t3). Though not fully comparable (as they used the Dimensional Obsessive-Compulsive Scale Short-Form, DOCS-SF), the results of Grøtte et al. (2022) —27.8% (in April 2020) and 24.0% (in December 2020)—are similar to ours. Although only assessed in a subsample (*n* = 430) in 2014, only 11% of the participants in the pre-pandemic period scored above the clinical cut-off. Grøtte et al. (2022) did not have access to pre-pandemic data but used retrospective assessments as an estimate, reporting a much smaller percentage of 2.33% above the cut-off score. Differences between the two studies may be explained by the measure (DOCS vs. OCI-R) and the point in time (2014 vs. retrospectively in 2020) when C-OCS was measured. Still, both studies point to an increase at the start of the pandemic, which was likely caused by an increase of ritualized washing in the general population when handwashing protocols were strongly advocated. Thus, the initial high rates of C-OCS may have represented adherence to hygiene protocols, thus overestimating pathological behavior. Once the focus of hygiene recommendations shifted toward wearing masks and social distancing, handwashing behavior decreased on average. Despite this decrease, the percentage of clinically relevant C-OCS was still elevated 12 months after the start of the pandemic.

Factors such as age, educational level, and baseline scores of obsessive compulsive symptom have been suggested to predict of a long-term course of obsessive compulsive symptoms (Abba-Aji et al., 2020; Fontenelle et al., 2021; Jelinek et al., 2021a; Alonso et al., 2021). Using these factors to predict change in C-OCS, C-OCS at the start of the pandemic (t1) was the best predictor (β = 0.56) and explained the largest amount of variance (∆*R*^2^ = 0.318). This corresponds with the findings of Grøtte et al. (2022). Hierarchical regression, showed that above the mentioned established predictors, (1) an increase in the duration (not the frequency) of handwashing and (2) an increase in negative reinforcement (distress reduction) served as additional independent predictors for the longitudinal increase in C-OCS. Our finding on the change in duration (not the frequency) of handwashing fits cross-sectional studies (Samuels et al., 2021) emphasizing the use of subjective criteria (Wahl et al., 2008) and the inability to terminate hygiene rituals in C-OCD (Hinds et al., 2012). It is also in line with Dean and Purdon (2021) reporting prolongation but not repetition of handwashing after contamination.

However, we acknowledge that the context, as well as social norms, differed between our study (during a pandemic) and the prior work (not during a pandemic). Accordingly, the impact of handwashing on OCS may vary. Still, the available longitudinal study extends these findings, implying a relationship between the decline in duration of handwashing (over a 3-month interval) and the long-term decrease in OCS. While the impact on the C-OCS was small (β = 0.069; ∆*R*^2^ = 0.005), these findings correspond to the results in Grøtte et al.’s (2022) study, which predicted the course of OCS based on “adherence to COVID-19 guidelines” (which included hand hygiene) in April 2020.

Besides handwashing, we also assessed distress reduction associated with handwashing. The decrease in distress after handwashing over the first 3 months of the pandemic and thus its distress-reducing effect served as an additional predictor of the long-term increase of OCS. Although the impact was small (β = 0.080; ∆*R*^2^ = 0.006) and should therefore be interpreted with caution, this result provides insight into an aspect of the topic that has received little attention to date. Currently, this aspect is given no weight in the discussion of detrimental effects of the pandemic measures but is in line with behavioral models of OCD (Dollard and Miller, 1950; Salkovskis, 1999), which are the basis for the assessment and treatment of OCD and are accepted all over the world. Still, nonexperimental empirical evidence in humans is scarce (Gagné et al., 2018).

The results were confirmed when we corrected for missing data (using multiple imputation), underlining their robustness. However, in the total model, only 34% of the variance was explained, emphasizing that it is a multifaceted process with other contributing factors [e.g., intolerance of uncertainty (Wheaton et al., 2021; Inozu et al., 2022), higher fear/anxiety (Ji et al., 2020), [pandemic-related] stress (Robillard et al., 2020), sleep disturbances (Cox and Olatunji, 2021), trait compulsivity (Albertella et al., 2021)]. Moreover, the OCI-R washing score at t1 accounted for 32% of the variance and less than an additional 1% of the variance was each accounted for by the change in hygiene behavior and change in negative reinforcement from t1 to t2. Thus, despite results from experimental work (Deacon and Maack, 2008) showing an increase in contamination fears after an increase in safety behavior (including hand hygiene) and the suggested distress-reducing effect of (ritualized) handwashing behavior (Shafran et al., 2020; Ornell et al., 2021), we did not find a general long-term increase in C-OCS in all participants. An elevated level of C-OCS at the start of the pandemic was the most important predictor for a decrease of C-OCS over time, which might be (partly) due to regression to the mean. Generally, our results further support that baseline symptomatology [including pre-pandemic symptoms (see Jelinek et al., 2021a; Grøtte et al., 2022)] are important to be considered (Alonso et al., 2021; Fontenelle et al., 2021).

No study comes without limitations. Those of the current study are as follows. First, generalizability may be limited in our study as only 56% of participants at t1 participated in the follow-up 12 months later. However, while on average participants were older and included fewer women and fewer participants with an A-level degree, participants did not differ from nonparticipants regarding psychopathology at t1. Moreover, the usual overrepresentation of women in online studies (Qiu et al., 2020) does not apply here. However, the sample size of 1,220 participants still offers the potential of robustness. Second, participants were not assessed in person. This was necessary to make the research possible during the ongoing pandemic and to ensure participant safety. Additionally, we used cookies to prevent multiple entries by the same participant and excluded persons with systematic response patterns. Still, video-or telephone-based assessments should be considered in future studies. Moreover, we were investigating obsessive compulsive symptoms, not criteria for a clinical diagnosis. To derive assumptions on the development of psychopathology, clinicians’ assessments would be necessary.

To summarize, hygiene behavior is important in health care, particularly during a pandemic. The WHO states in a healthcare recommendation: “Hand hygiene is therefore the most important measure to avoid the transmission of harmful germs and prevent health care-associated infections”(WHO, 2022, p. 1). However, considering *how* these recommendations are communicated to the general population seems vital to prevent side effects such as an increase in C-OCS. Although effects were small, first, increase in the duration of handwashing predicted a long-term increase in OCS. Accordingly, in addition to the exact procedures for handwashing, specifications for the duration of handwashing (20–30 s) are necessary. These specifications are included in the recommendations of healthcare organizations, (e.g., WHO, NHS) and should not be omitted to prevent the usage of subjective measures (“until my hands feel clean”). Secondly, an increased “calming effect” was associated with the long-term course of OCS. Again only with a small effect size, but clear instructions may be helpful regarding when hands should be washed (e.g., before eating or preparing food, after going to the bathroom, upon returning home). These recommendations need to be tested in interventional trials.

In summary, the present study supports an increase in C-OCS in the general population from the pre-pandemic period to the first few months of the COVID-19 pandemic. Over the first year of the pandemic, however, OCS decreased. The course of C-OCS over the first year of the pandemic was primarily predicted by C-OCS at the start of the pandemic. An effect of the duration of handwashing and a distress-reducing effect of handwashing were ascertainable but small. To understand the long-term trajectory of OCS as well as predictors of its course, studies with longer assessment periods are necessary.

## Author’s note

For a previous publication on the OCS data of this sample at t1 and t2, see Jelinek et al. (2021a).

## Data availability statement

The raw data supporting the conclusions of this article will be made available by the authors, without undue reservation.

## Ethics statement

The studies involving humans were approved by Approval was granted by the Local Psychological Ethics Committee (LPEK, #LPEK-0129, #LPEK-0298). The studies were conducted in accordance with the local legislation and institutional requirements. The participants provided their written informed consent to participate in this study.

## Author contributions

LJ: Conceptualization, Data curation, Formal analysis, Investigation, Methodology, Project administration, Resources, Software, Validation, Visualization, Writing – original draft, Writing – review & editing. AG: Conceptualization, Investigation, Methodology, Project administration, Resources, Writing – review & editing. FM: Conceptualization, Investigation, Methodology, Project administration, Writing – review & editing. LS: Formal Analysis, Writing – review & editing. SM: Conceptualization, Investigation, Methodology, Project administration, Resources, Writing – review & editing. AY: Conceptualization, Methodology, Resources, Writing – review & editing. JM: Conceptualization, Investigation, Methodology, Project administration, Writing – review & editing.
