# Supplementary material for: Too much of a good thing? Hand hygiene and the long-term course of contamination-related obsessive-compulsive symptoms
Source: Front Psychol. 2024 Mar 8;15:1279639. doi: 10.3389/fpsyg.2024.1279639 (PMC10959097; doi:10.3389/fpsyg.2024.1279639)
Supplement: Supplementary file 1 [file Data_Sheet_1.docx]

**Online-Only Supplements**

**eTable 1.** Predictors of Change in Contamination-related Obsessive-Compulsive Symptoms (Outcome: Change in OCI-R washing subscale from t1 to t3), *n* = 852, Steps 3 and 5 Switched

**eTable 2.** Sensitivity Analysis: Predictors of Change in Contamination-related Obsessive-Compulsive Symptoms (Outcome: Change in OCI-R Washing Scale from t1 to t3), *n* = 1220; Step Steps 3 and 5 Switched

**eTable 1.** Predictors of Change in Contamination-related Obsessive-Compulsive Symptoms (Outcome: Change in OCI-R washing subscale from t1 to t3), *n* = 852, Steps 3 and 5 Switched

|  | ***B* [*CI_95%_*]** | **β** | ***p*** |
| --- | --- | --- | --- |
| **Step 1** | **Model B1** | | |
| Constant | 1.531 [−0.721, 2.341] |  | < 0.001 |
| Age | − 0.014 [−0.027, −0.002] | −0.081 | 0.022 |
| Gender^a^ | −0.112[−0.434, 0.209] | −0.024 | 0.493 |
| A-levels | −0.027[−0.348, 0.295] | −0.006 | 0.870 |
| **Step 2** | **Model B2** | | |
| Constant | −0.003 [−0.688, 0.682] |  | 0.993 |
| Age | −0.009 [−0.019, 0.002] | −0.048 | 0.096 |
| Gender^a^ | −0.070 [−0.335, 0.196] | −0.015 | 0.606 |
| A-levels^b^ | −0.069[−.334, 0.196] | −0.015 | 0.611 |
| OCI−R washing subscale (t1) | 0.515 [0.464, 0.565] | 0.565 | < 0.001 |
|  |  |  |  |
| χ²(1) = 402.51, *p* < 0.001 |  |  |  |
| **Step 3** | **Model B3** | | |
| Constant | −0.031 [−0.713, 0.651] |  | 0.929 |
| Age | −0.008 [−0.019, 0.002] | −0.048 | 0.101 |
| Gender^a^ | −0.072 [−0.336, 0.192] | −0.015 | 0.591 |
| A-levels^b^ | −0.045 [−0.309, 0.219] | −0.010 | 0.738 |
| OCI−R washing subscale (t1) | 0.512 [0.462, 0.563] | 0.562 | > 0.001 |
| Change in negative reinforcement after handwashing (t1–t2) | 0.206 [0.069, 0.343] | 0.083 | 0.003 |
|  |  |  |  |
| χ²(1) = 8.73, *p* = 0.003 |  |  |  |
| **Step 4** | **Model B4** |  |  |
| Constant | -0.185 [−0.878, 0.508] |  | 0.601 |
| Age | -0.008 [−0.018, 0.002] | −0.046 | 0.109 |
| Gender^a^ | -0.069 [−0.333, 0.194] | −0.015 | 0.607 |
| A-levels^b^ | -0.023 [-0.287, 0.241] | −0.005 | 0.864 |
| OCI−R washing subscale (t1) | 0.514 [0.463, 0.564] | 0.564 | > 0.001 |
| Change in negative reinforcement after handwashing (t1–t2) | 0.198 [0.061, 0.335] | 0.080 | 0.005 |
| Change in duration of handwashing (t1–t2) | 0.148 [0.022, 0.273] | 0.065 | 0.021 |
|  |  |  |  |
| χ²(1) = 84.72, *p* = 0.030 |  |  |  |
| **Step 5** | **Model B5** | | |
| Constant | -0.187 [−0.884, 0.510] |  | 0.598 |
| Age | -0.008 [−0.018, 0.002] | −0.046 | 0.109 |
| Gender^a^ | -0.069 [−0.333, 0.195] | −0.015 | 0.609 |
| A-levels^b^ | -0.023 [-0.288, 0.241] | −0.005 | 0.863 |
| OCI−R washing subscale (t1) | 0.514 [0.463, 0.564] | 0.564 | < 0.001 |
| Change in negative reinforcement after handwashing (t1–t2) | 0.198 [0.060, 0.335] | 0.080 | 0.005 |
| Change in duration of handwashing (t1–t2) | 0.147 [0.021, 0.274] | 0.065 | 0.023 |
| Change in frequency of handwashing (t1–t2) | 0.003 [−0.088, 0.094] | 0.002 | 0.950 |
|  |  |  |  |
| χ²(1) < 0.001, *p* = 0.990 |  |  |  |

*Notes. R²* = .008, *F* = 2.216 (*p =* .085) for step 1; ∆*R²* = .318, *F* = 102.140 (*p <* .001) for step 2; ∆*R²* = .007, *F* = 84.199 (*p* < .001) for step 3; ∆*R²* = .004, *F* = 71.410 (*p* > .001) for step 4; ∆*R²* < .001, *F* = 61.137 (*p* > .001) for step 5. *b* = unstandardized regression coefficient, β = standardized regression coefficient, χ² = Chi-Square test statistic for model comparison to the previous step via LRT. OCI-R = Obsessive-Compulsive Inventory-Revised; a 1 = male, 2 = female, b 0 = no A-level, 1 = A-level and above

**eTable 2.** Results Using Multiple Imputation: Predictors of Change in Contamination-related Obsessive-Compulsive Symptoms (Outcome: Change in OCI-R Washing Scale from t1 to t3), *n* = 1220; Steps 3 and 5 Switched

|  | ***B* [*CI_95%_*]** | **β** | ***p*** |
| --- | --- | --- | --- |
| **Step 1** | **Model B1** | | |
| Constant | 1.034 [0.434, 1.633] |  | .001 |
| Age | - 0.008 [−0.018, 0.001] | −0.049 | .096 |
| Gender^a^ | -0.218 [-0.487, 0.051] | -0.046 | .112 |
| A-levels | 0.088 [-0.182, 0.358] | 0.019 | .525 |
|  |  |  |  |
| **Step 2** | **Model B2** | | |
| Constant | -0.581 [−1.101, -0.060] |  | .029 |
| Age | -0.001[−0.009, 0.007] | -0.005 | .839 |
| Gender^a^ | -0.185 [−0.410, 0.041] | -0.039 | .108 |
| A-levels^b^ | 0.102 [-0.124, 0.328] | -0.022 | .378 |
| OCI−R washing subscale (t1) | 0.502 [0.459, 0.546] | 0.547 | > .001 |
|  |  |  |  |
| χ²(1) = 433.57, *p* < 0.001 |  |  |  |
| **Step 3** | **Model B3** | | |
| Constant | -0.617 [−1.137, -0.097] |  | .020 |
| Age | -0.001 [−0.009, 0.008] | -0.003 | .893 |
| Gender^a^ | -0.195 [−0.420, 0.030] | -0.042 | .089 |
| A-levels^b^ | 0.123 [-0.103, 0.349] | 0.026 | .285 |
| OCI−R washing subscale (t1) | 0.500 [0.457, 0.543] | 0.545 | > .001 |
| Change in negative reinforcement after handwashing (t1-t2) | 0.193 [0.066, 0.319] | 0.079 | .003 |
|  |  |  |  |
| χ²(1) = 8.82, p = 0.003 |  |  |  |
| **Step 4** | **Model B4** | | |
| Constant | -0.732 [−1.295, -0.233] |  | .007 |
| Age | -0.001 [−0.009, 0.008] | -0.004 | .869 |
| Gender^a^ | -0.184 [−0.408, 0.041] | -0.039 | .109 |
| A-levels^b^ | 0.132 [-0.094, 0.357] | 0.028 | .252 |
| OCI−R washing subscale (t1) | 0.503 [0.460, 0.546] | 0.548 | > .001 |
| Change in negative reinforcement after handwashing (t1–t2) | 0.185 [0.059, 0.311] | 0.076 | .004 |
| Change in duration of handwashing (t1–t2) | 0.134 [0.023, 0.246] | 0.063 | .019 |
|  |  |  |  |
| χ²(1) = 5.537, *p* = 0.019 |  |  |  |
| **Step 5** | **Model B5** |  |  |
| Constant | -0.764 [−0.884, 0.510] |  | 0.005 |
| Age | -0.001 [−0.009, 0.008] | −0.004 | 0.878 |
| Gender^a^ | -0.182 [−0.406, 0.043] | −0.039 | 0.609 |
| A-levels^b^ | 0.128 [-0.097, 0.354] | 0.027 | 0.863 |
| OCI−R washing subscale (t1) | 0.504 [0.461, 0.548] | 0.549 | < 0.001 |
| Change in negative reinforcement after handwashing (t1–t2) | 0.180 [0.053, 0.306] | 0.060 | 0.005 |
| Change in duration of handwashing (t1–t2) | 0.127 [0.014, 0.239] | 0.065 | 0.028 |
| Change in frequency of handwashing (t1–t2) | 0.045 [−0.044, 0.132] | 0.028 | 0.322 |
|  |  |  |  |
| χ²(1) = 0.989, *p* = 0.320 |  |  |  |

*Notes. R²* = 0.006., *F* = 2.481 (*p =* 0.059) for step 1; ∆*R²* = 0.297, *F* = 132.275 (*p <* .001) for step 2; ∆*R²* = 0.007, *F* = 107.860 (*p* < .001) for step 3; ∆*R²* = 0.004., *F* = 90.692 (*p* > .001) for step 4; ∆*R²* = 0.001., *F* = 77.769 (*p* > .001) for step 5. *b* = unstandardized regression coefficient, β = standardized regression coefficient, χ² = Chi-Square test statistic for model comparison to the previous step via LRT. OCI-R = Obsessive-Compulsive Inventory-Revised; ^a^ 1 = male, 2 = female, ^b^ 0 = no A-level, 1 = A-level and above
